# A novel regulator RcdA of the *csgD* gene encoding the master regulator of biofilm formation in *Escherichia coli*

**DOI:** 10.1002/mbo3.42

**Published:** 2012-10-08

**Authors:** Tomohiro Shimada, Yasunori Katayama, Shuichi Kawakita, Hiroshi Ogasawara, Masahiro Nakano, Kaneyoshi Yamamoto, Akira Ishihama

**Affiliations:** 1 Department of Frontier Bioscience and Research Center for Micro-Nano Technology, Hosei UniversityKoganei, Tokyo, 184-8584, Japan; 2 Chemical Resources Laboratory, Tokyo Institute of TechnologyNagatsuda, Yokohama, 226-8503, Japan; 3 Research Center for Human and Environmental Sciences, Shinshu UniversityUeda, Nagano, 336-8567, Japan

**Keywords:** Biofilm formation, *csgD* promoter, genomic SELEX, regulation network, transcription factor

## Abstract

The FixJ/LuxR family transcription factor CsgD is a master regulator of biofilm formation in *Escherichia coli*. Previously, we identified more than 10 transcription factors that participate in regulation of the *csgD* promoter. After genomic SELEX screening of regulation targets, an uncharacterized TetR-type transcription factor YbjK was found to be involved in regulation of the *csgD* promoter. In addition, a number of stress-response genes were found to be under the direct control of YbjK. Taken together, we propose to rename it to RcdA (regulator of *csgD*). One unique feature of RcdA is its mode of DNA binding. Gel shift, DNase-I footprinting, and atomic force microscopic (AFM) analyses indicated that RcdA is a DNA-binding protein with a high level of cooperativity, with which it covers the entire surface of probe DNA through protein–protein interaction and moreover it induces the formation of aggregates of DNA–RcdA complexes.

## Introduction

When *Escherichia coli* cells switch the life mode from single planktonic cell growth to biofilm mode, the flagella formation is turned off and in turn the production of curli fimbriae and extracellular polysaccharides for cell–cell adhesion is switched on, thereby leading to biofilm formation (Barnhart and Chapman 2006; Lopez et al. 2011). In *E. coli*, the motility for planktonic growth is under the positive control by three stages of a regulatory forward cascade including the master regulator FlhDC at the first stage and RpoF (or FliA) sigma factor at the second stage (Claret and Hughes 2002; Stafford et al. 2005). In contrast, the biofilm formation is under the positive control by another regulatory cascade including the master regulator CsgD and RpoS-RpoE sigma factors (Gerstel et al. 2003; Beloin et al. 2004; Gualdi et al. 2007). The FixJ/LuxR family protein CsgD is a key regulator for curli production, and modulates the expression of not only the *csg* operon encoding components and assembly of curli (Hammar et al. 1995), but also a set of genes for adaptation of cell physiology to the biofilm lifestyle (Chirwa and Herrington 2003; Brombacher et al. 2006). ChIP-chip analysis of CsgD-bound DNA sequences in vivo indicated a total of approximately 30 regulation targets that are mostly involved in stress response, altogether forming another regulatory cascade including several transcription factors (Ogasawara et al. 2011). Among the regulation targets of CsgD, we identified the *flhDC* operon encoding the master regulator of flagella formation, FlhDC, indicating the antagonistic cross-talk between biofilm formation and flagella formation at the top of two alternative hierarchic networks of regulation toward planktonic single-cell growth and biofilm cell assemblies (Pesavento et al. 2008; Ogasawara et al. 2011).

In good agreement with the master regulator function of CsgD, transcription of *csgD* is under the control of more than 10 transcription factors, each monitoring a specific and different factor or condition in environment (Ishihama 2010; Ogasawara et al. 2010a,b). In the course of genomic SELEX screening of regulation targets by a total of approximately 300 species of transcription factors from *E. coli*, we identified YbjK, a hitherto uncharacterized TetR-type transcription factor, which is involved in regulation of a number of genes that are involved in biofilm formation, including the *csgD* operon. In addition, YbjK was found to regulate a variety of stress-response genes. One unique feature of YbjK is its strong cooperative DNA-binding mode, leading to aggregation of YbjK–DNA complexes as observed with gel shift, DNase footprinting, and atomic force microscopic (AFM) analyses. After detailed analysis of the functional roles and molecular properties, we propose that YbjK is a novel regulator of biofilm formation located on the top of hierarchic regulation network, and then propose to rename it as RcdA (regulator of *csgD*).

## Experimental Procedures

### Bacterial strains and culture conditions

*Escherichia coli* BL21(DE3) (F- *ompT hsd* [rB- mB-] *dcm gal* λ[DE3]) (Studier and Mofatt 1986) was used for expression and purification of RcdA. *Escherichia coli* DH5α (F *lacZ* M15 *recA endA hsdR phoA supE*44 *thi gyrA relA*) was used for cloning sequencing of SELEX fragments (see below for SELEX screening system). *Escherichia coli* K-12 BW25113 (*lacI*^q^ *rrnB lacZ hsdR araBAD rhabAD*) and its otherwise isogenic mutant strain JW5114 lacking the *rcdA* gene were products of the Keio collection (Baba et al. 2006), and obtained from National Bio-Resource Center (National Institute of Genetics, Japan). *Escherichia coli* MC4100 (Casadaban 1976) was used for construction of the promoter–*lacZ* reporter fusion vectors (see below). Cells were cultured in LB medium or YESCA medium (Pratt and Silhavy 1998). When necessary, 100 μg/mL ampicillin and 50 μg/mL kanamycin were added in to the medium.

### Plasmid construction

For construction of RcdA-expression plasmid, pRcdA, for its expression, a DNA fragment corresponding to the *rcdA*-coding region was amplified by polymerase chain reaction (PCR) using *E. coli* K-12 W3350 genome DNA as a template, and after digestion with *NdeI* and *Not*I within the primer sequences, cloned into pET21a(+) (Novagen, Darmstadt, Germany) at the corresponding sites. For the construction of *lacZ* reporter vectors, DNA fragments, each containing the RcdA target promoter region, were prepared by PCR using *E. coli* K-12 W3350 genome DNA as a template and a pair of gene-specific primers. After digestion with *Eco*RI and *Bam*HI, the PCR-amplified fragments were inserted into pRS551 (Simons et al. 1987) at the corresponding sites to generate the promoter assay vectors.

### Expression and purification of RcdA protein

His-tagged RcdA was expressed under the control of the T7 promoter in pRcdA-transformed *E. coli* BL21(DE3) and affinity purified according to the standard purification procedure (Shimada et al. 2005; Yamamoto et al. 2005).

### Production of anti-RpoA and anti-RcdA antibodies

Polyclonal anti-RpoA and anti-RcdA antibodies were produced in rabbits after injection of purified RpoA (His-tag-free form) and purified His-tag RcdA protein, respectively.

### Genomic SELEX screening for RcdA-binding sequences

The improved genomic SELEX system was as described previously (Shimada et al. 2005). A mixture of DNA fragments of the *E. coli* K-12 W3110 genome was prepared after sonication of purified genome DNA, and cloned into a multicopy plasmid pBR322. In each SELEX screening, the DNA mixture was regenerated by PCR. For SELEX screening, 5 pmol of the mixture of DNA fragments and 10–20 pmol His-tagged RcdA were mixed in a binding buffer (10 mmol/L Tris-HCl, pH 7.8 at 4°C, 3 mmol/L magnesium acetate, 150 mmol/L NaCl, and 1.25 mg/mL bovine serum albumin) and incubated for 30 min at 37°C. The DNA–RcdA mixture was applied to a Ni-NTA column, and after washing out unbound DNA with the binding buffer containing 10 mmol/L imidazole, DNA–RcdA complexes were eluted with an elution buffer containing 200 mmol/L imidazole. The sequences of DNA fragments were determined by SELEX-clos and SELEX-chip methods (Ishihama 2010; Shimada et al. 2011a,b).

For SELEX-clos (cloning-sequencing) analysis, DNA fragments recovered from the DNA–RcdA complexes were purified, PCR-amplified, cloned into pT7 Blue-T vector (Novagen), and transformed into *E. coli* DH5α. Sequencing of each clone was carried out using the T7 primer (5′-TAATACGACTCACTATAGGG-3′) (Shimada et al. 2005). For SELEX-chip analysis, PCR-amplified genomic SELEX-DNA fragments were labeled with Cy5 while the original DNA library was labeled with Cy3. The mixture of fluorescent-labeled DNA samples was hybridized with a DNA microarray consisting of 43,450 species of 60-bp-long DNA probes, which are designed to cover the entire *E. coli* genome at 105-bp intervals (Oxford Gene Technology, Oxford, U.K.) (Shimada et al. 2008; Teramoto et al. 2010). The fluorescent intensity of test sample at each probe was normalized with that of the corresponding peak of original library. After normalization of each pattern, the Cy5/Cy3 ratio was measured and plotted against the corresponding position along the *E. coli* genome.

### Gel-shift assay of RcdA–DNA complexes

Probes, each carrying the promoter region, were generated by PCR amplification of using a pair of primers (5′-FITC-labeled Lac30R-FITC and 551SQ-F), plasmids carrying SELEX fragments (100 ng) as the templates, and Ex Taq DNA polymerase. All the PCR products with FITC at 5′ termini were purified by polyacrylamide gel electrophoresis, and then used for gel-shift assay under the standard conditions (Ogasawara et al. 2007a,b).

### DNase-I footprinting assay

DNase-I footprinting assay was carried out under the standard reaction conditions (Ogasawara et al. 2007a,b). In brief, 1.0 pmol each of FITC-labeled probes was incubated at 37°C for 30 min with various concentrations of purified RcdA in 25 μL of 10 mmol/L Tris-HCl (pH 7.8), 150 mmol/L NaCl, 3 mmol/L magnesium acetate, 5 mmol/L CaCl_2_, and 25 mg/mL BSA. After incubation for 30 min, DNA digestion was initiated by adding 5 ng DNase I (Takara, Otsu, Japan). After digestion for 30 sec at 25°C, the reaction was terminated by the addition of 25 μL phenol. DNA was precipitated from aqueous layer by ethanol, dissolved in formamide dye solution, and analyzed by electrophoresis on a 6% polyacrylamide gel containing 8 mol/L urea.

### Measurement of the promoter activity

The test promoter–*lacZ* fusion plasmid was transformed into *E. coli* MC4100 (Casadaban 1976) and the transformant was used as the host for preparation of λRS45. The recombinant phage containing promoter–*lacZ* fusion was isolated from the resulting phage lysate and infected onto *E. coli* BW25113 and *E. coli* BW*rcdA* for screening of kanamycin-resistant and Lac+ colonies. Single-copy promoter–*lacZ* fusion strains were grown in YESCA medium and subjected to measuring β-galactosidase activity with *o*-nitrophenyl-d-galactopyranoside as described by Miller (1972).

### Northern blot analysis

Northern blot analysis was performed using fluorescence-labeled probes under the standard reaction conditions (Ogasawara et al. 2010a,b). Total RNAs were extracted from exponentially growing *E. coli* cells (OD_600_ = 0.5) by the hot phenol method. RNA purity was checked by electrophoresis on 2% agarose gel in the presence of formaldehyde followed by staining with ethidium bromide. Fluorescent-labeled probes were prepared by PCR amplification using W3110 genomic DNA (50 ng) as template, DIG-11-dUTP (Roche, Almere, the Netherlands) and dNTP as substrates, gene-specific forward and reverse primers (for primer sequences see Table 1), and Ex Taq DNA polymerase (Takara). Total RNAs (4 μg) were incubated in formaldehyde-MOPS (morpholinepropanesulfonic acid) gel-loading buffer for 10 min at 65°C for denaturation, subjected to electrophoresis on formaldehyde-containing 2% agarose gel, and then transferred to nylon membrane (Roche). Hybridization was performed with DIG easy Hyb system (Roche) at 50°C overnight with a DIG-labeled probe. For detection of the DIG-labeled probe, the membrane was treated with anti-DIG-AP Fab fragments and CDP-Star (Roche), and the image was scanned using LAS-4000 IR multicolor (Fuji Film, Tokyo, Japan).

**Table 1:** RcdA target list (SELEX-clos)

Function	Left		S		Right	Function	No.
	*tfaX*	>		>	*appY*	Transcription factor	22
Predicted transporter	*ybjJ*	<		>	*rcdA*	Transcription factor	15
BLUE-EAL antirepressor	*ycgF*	<		>	*ycgZ*	RcsAB connector	6
SOS cell division inhibitor	*sulA*	<		>	*sxy*	CRP coactivator	3
	*pinE*	>		>	*mcrA*	Restriction enzyme	2
	*gtrB*	>		>	*gtrS*	Glucosyl transferase	2
CP4-6 conserved protein	*yagK*	<		<	*yagL*		2

Genomic SELEX screening of RcdA-binding sequences was performed under the standard conditions (for details see text). RcdA-bound DNA fragments were subjected to SELEX-clos analysis. The clones independent obtained more than twice are shown (No., number of clones). All these fragments are located within spacer regions between “Right” and “Left” genes. The direction of transcription is shown by arrows.

### Western blotting assay

Intracellular concentrations of RpoA and RcdA were determined by using the Western blotting assay under the standard conditions (Jishage and Ishihama 1995). In brief, *E. coli* whole lysates were prepared by sonication after lysozyme treatment, and directly subjected to SDS-PAGE. After transfer proteins onto filters, the protein-blotted filter was treated with anti-RcdA antibody, which was raised in rabbits against purified RcdA. Antibodies were detected with fluorescent-labeled mouse anti-rabbit IgG antibody. RpoA level was measured as an internal reference for determination of the RcdA level.

### Biofilm assay

For quick determination of biofilm formation, Crystal violet staining method was employed (Sule et al. 2009). In short, *E. coli* cells were grown in either LB or YESCA medium and both at 28 and 37°C using 96-well microplate. After incubation for various times, planktonic cells were discarded and plate was washed twice with PBS(−) and then stained with 0.1% Crystal violet for 20 min at room temperature. After extensive washing with H_2_O, biofilm-bound Crystal violet was extracted with 70% ethanol and measured at OD 600 nm.

The level of biofilm formation was also confirmed by staining agar plate-grown *E. coli* with Congo red under the standard conditions (Ogasawara et al. 2010a). In brief, each *E. coli* strain was grown at 28°C for 48 h on YESCA plates containing 50 μg/mL Congo red and 10 μg/mL Commassie blue.

### AFM observation

For AFM observation of RcdA–DNA complexes, mixtures of 496-bp-long *rcdA* promoter DNA and various concentrations of RcdA protein in a binding buffer (50 mmol/L HEPES-KOH [pH 7.6], 1 mmol/L EDTA, 500 mmol/L NaCl, 1 mmol/L DTT) were incubated for 1 h at 37°C. After cross-linking DNA–protein by treatment with 0.1% glutaraldehyde for 30 min at 4°C, the samples were diluted to make the final DNA concentration approximately 5 ng/μL with a dilution buffer (5 mmol/L HEPES-KOH [pH 7.6], 2 mmol/L MgCl_2_) and directly spotted onto a freshly cleaved mica substrate pretreated with 10 mmol/L spermidine. After 10 min, the mica was gently washed with distilled water and dried under nitrogen gas. Imaging was performed in Tapping Mode™ using a Multimode™ AFM (Veeco, Santa Barbara, California) operation using a Nanoscope IIIa™ controller. For scanning, Olympus silicon cantilevers (OMCL-AC160TS-W2) with spring constants between 36 and 75 N/m were used. The scan frequency was typically 1.5 Hz per line and the modulation amplitude was a few nanometers. Analysis of the DNA–YcdA complexes was performed using the Nanoscope software. Two-dimensional images in brightness and contrast were optimized for the purpose of clarity. Three-dimensional images were created using the Nanoscope software and exported in TIFF format.

## Results

### Genomic SELEX screening of RcdA-binding sequences: SELEX-clos

For the identification of DNA sequences that are recognized by *E. coli* RcdA, we employed the genomic SELEX screening system (Shimada et al. 2005), in which a library of *E. coli* genome DNA fragments 200–300 bp in length was used for screening of DNA sequences with RcdA-binding activity instead of synthetic oligonucleotides with all possible sequences used in the original SELEX method (Ellington and Szostak 1990; Tuerk and Gold 1990; Singer et al. 1997). Into the mixture of *E. coli* genomic DNA fragments, two- or fourfold molar excess of the purified His-tagged RcdA protein was added, the RcdA–DNA complexes formed were affinity purified. In the early stage of this genomic SELEX cycle, the RcdA-bound DNA fragments gave smear bands on PAGE as did the original genome fragment mixture. After two and three SELEX cycles, DNA fragments with high affinity to RcdA were enriched, forming discrete bands on PAGE.

**Figure 1:**
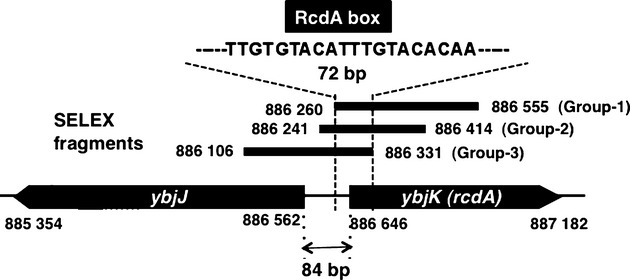
RcdA-binding sequences identified by SELEX-clos. Genomic SELEX screening of RcdA-binding sequences was performed using purified His-tagged RcdA and a collection of *Escherichia coli* genome DNA fragments. DNA fragments associated with RcdA were subjected to SELEX-clos (cloning and sequencing). DNA fragments isolated more than twice are listed in Table 1. A total of 22 clones carried segments from the spacer between *ybjJ* and *rcdA*, which can be classified into three groups (Group-1, Group-2, and Group-3). Among the three groups, a sequence of 72 bp in length overlapped, which included the putative palindromic RcdA-box sequence. The numbers shown in each segment represent the genome position of *E. coli* K-12 W3110 (PEC database, NIG, Msihima, Japan).

For identification of the sequences of DNA fragments bound to RcdA, we first performed SELEX-clos (cloning-sequencing) analysis. A total of 82 SELEX fragments were cloned and sequenced, of which 52 were cloned more than twice whereas 30 were identified only once (Table 1). The most abundant clones (22 clones) carried the spacer region sequence upstream of *appY* encoding transcription factor for a group of genes involved in purine degradation on one direction and downstream of *tfaX* on another direction. Sequence analysis indicated that three different groups of the RcdA-binding fragments were included in these 22 clones (Fig. 1). A segment 72 bp in length overlapped between these three group fragments, suggesting that the RcdA-binding site is located within this 72-bp-long short segment. In this 72-bp fragment, a palindromic sequence of TTGTGTACAttTGTACACAA was identified, which may be the binding site of RcdA (see below for the RcdA box sequence). A total of 15 clones carried the spacer sequence between divergently organized *rcdA* itself and *ybjJ* encoding a major facilitator superfamily (MFS) transporter. YbjJ is predicted to be involved in microbial drug efflux (Saidijam et al. 2006). Six clones were isolated from the spacer between *ycgF* (the blue light-responsive regulator with EAL domain) and *ycgZ* (the connector protein for RcsB regulation of biofilm formation). Three clones were isolated from the spacer between *sulA* (SOS cell division inhibitor) and *sxy* (CRP coactivator). Two clones each were isolated from three intergenic spacer regions: upstream of *mcrA* encoding a restriction enzyme; upstream of *gtrS* encoding glucosyl transferase; and upstream of *yagK* encoding a conserved CP4-6 prophage protein. All these genes were predicted to be under the direct control of RcdA. Noteworthy is that all the genes with known functions are related to response to various stresses including biofilm formation.

SELEX-clos allows the screening of DNA sequences with high affinity to the test transcription factor, but it is not practical for identification of the whole set of its binding targets because the number of isolated clones correlates with the binding affinity as demonstrated for a variety of transcription factors such as Cra, CRP, and LeuO (Shimada et al., 2011a,b). For identification of the whole set of RcdA-binding sequences, we then subjected the mixture of SELEX fragments to the SELEX-chip analysis.

### Genomic SELEX screening of RcdA-binding sequences: SELEX-chip

**Figure 2:**
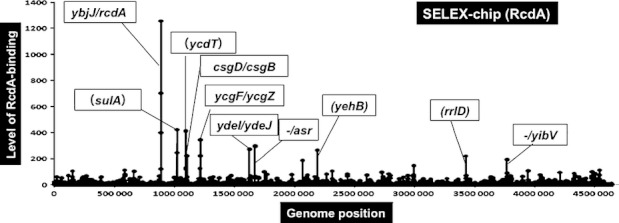
RcdA-binding sites on the *Escherichia coli* K-12 genome identified by SELEX-chip. After genomic SELEX screening of RcdA-binding sequences, a collection of DNA fragments was subjected to SELEX-chip analysis using the tilling array of *E. coli* K-12 genome. The *y*-axis represents the relative number of RcdA-bound DNA fragments, whereas the *x*-axis represents the genome position of *E. coli* K-12. The regulation targets were predicted based on the RcdA-binding sites. When RcdA binds within spacer regions, flanking genes on both sides are indicated. If the site is located downstream of the gene, it is indicated by minus symbol. The genes shown in parentheses represent RcdA-binding sites on open reading frames. The whole set of predicted regulation targets of RcdA are described in Table 2.

Genomic SELEX fragments were labeled with Cy5 whereas the original DNA library was labeled with Cy3. The mixture of fluorescent-labeled samples was hybridized to a DNA tilling microarray (Shimada et al. 2008; Teramoto et al. 2010). The ratio of fluorescence intensity bound to each probe between the test sample and the original library DNA was measured and plotted against the corresponding position along the *E. coli* genome (Fig. 2). On the DNA tilling array used, the 60-bp-long probes are aligned along the *E. coli* genome at 105-bp intervals, and therefore approximately 300-bp-long SELEX fragments should bind to two or more consecutive probes. The height of RcdA binding correlates with the affinity to the RcdA protein.

A total of 11 peaks were identified by setting the cutoff level at 100 (Fig. 2) and nine additional peaks by decreasing the cutoff level to 50 (Table 2). Among the total of 20 SELEX-chip peaks examined, nine were already identified by SELEX-clos (see Table 1). Based on the criteria that prokaryotic transcription factor-binding sites are located upstream of the regulation target genes (Ishihama 2010), a total of 27 genes were predicted to be the potential regulation targets of RcdA (see Tables 1 and 2). It is noteworthy that among these 27 predicted regulation targets of RcdA, six stress-response transcription regulators, AppY (the regulator of purine degradation operon), Sxy (coactivator for cAMP-CRP), CsgD (the master regulator of biofilm formation), YcgF (the blue light-responsive regulator with EAL domain), FimB (the regulator of *fim* switching), and RcdA itself, are all under the direct control of RcdA, indicating that a number of genes must be regulated indirectly through these five transcription factors. Within this regulation network, RcdA should be located at a higher level of the hierarchy.

**Table 2:** RcdA target list (SELEX-chip)

Function	Left		S		Right	Function
Hydroxymethyltransferase	*panB*	<	*yadC*	<	*yadK*	
	*tfaX*	>		>	*appY*	Transcription factor
Lipoate synthase	*lipA*	<	*ybeF*	<	*ybeF*	
Transporter	*ybjJ*	<		>	*rcdA*	Transcription factor
Outer membrane protein A	*ompA*	<	*sulA*	>	*sxy*	CRP-dependent promoters
Biofilm adhesin PGA export	*pgaA*	<	*ycdT*	<	*insE4*	
Transcription factor	*csgD*	<		>	*csgB*	Curlin nucleator protein
	*pinE*	>		>	*mcrA*	5-Met-C restriction nuclease B
Antirepressor for YcgE	*ycgF*	<		>	*ycgZ*	Connector for RcsB regulation
Conserved protein	*ydeI*	<		>	*ydeJ*	Conserved protein
	*ynfM*	>		>	*asr*	Acid shock periplasm prot
	*ydhS*	>	*ydhT*	<	*ydhU*	
	*insH6*	<		>	*yoeA*	Hemin or colicin receptor
Fimbrial-like adhesin protein	*yehA*	<	*yehB*	<	*yehC*	
	*micF*	>		>	*rcsD*	RcsBC TCS intermediate prot
	*pbl*	>	*ygeK*	<	*ygeL*	
5S rRNA of rrnD operon	*rrfD*	<	*rrlD*	<	*alaU*	
	*yibG*	>		>	*yibV*	Hypothetical protein
	*gltU*	>	*rrlC*	>	*rrfC*	5S rRNA of *rrnC* operon
N-AceNA OM channel protein	*nanC*	<		>	*fimB*	On/off regulator of *fimA*

Genomic SELEX screening of RcdA-binding sequences was performed under the standard conditions (for details see text). RcdA-bound DNA fragments were subjected to SELEX-chip analysis. The pattern of positions of RcdA-bound DNA fragments are shown in Figure 2. The peak positions of SELEX fragments are shown in “S” column. In some cases, RcdA-binding sites were identified on open reading frames on the indicated genes. The genes located on “Left” and “Right” sides are indicated. The direction of transcription is shown by arrows.

A group of membrane-associated stress-response proteins such as OmpA (OM protein 3a), YbjJ (transporter), PgaA (PGA transporter), NanC (Neu5Ac channel), YoeA (hemin receptor), CsgB (curlin fimbriae), and YehA (type-1 fimbrial protein) are also included as an abundant group in the list of predicted targets of RcdA. In addition, stress-response cytoplasmic proteins such as YdeI (hydrogen peroxide-response protein) and Asr (acid shock-response protein) are also predicted to be under the direct control of RcdA.

### Binding in vitro of RcdA to target sequences

**Figure 3:**
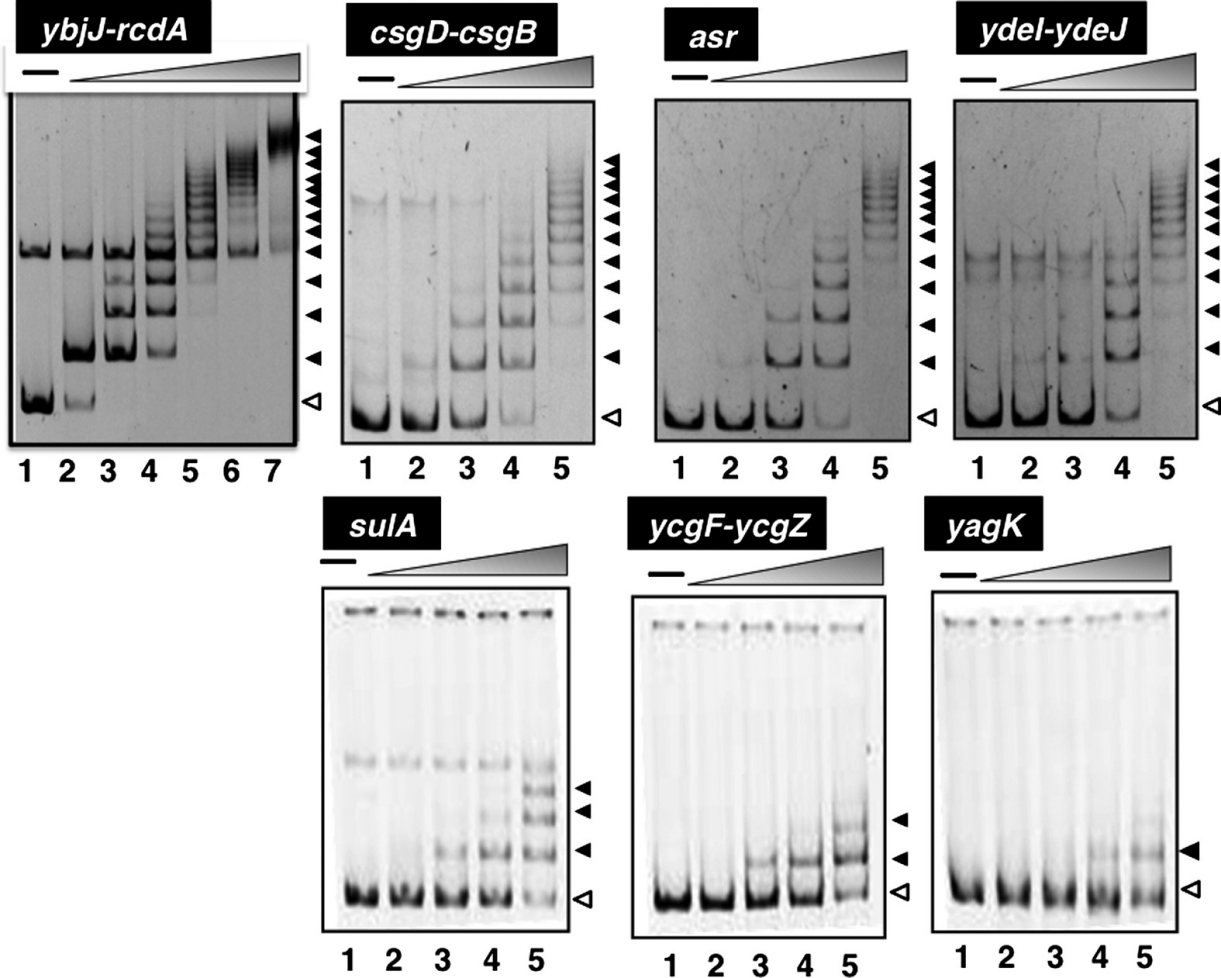
Gel-shift assay of RcdA binding to target DNAs. The RcdA-binding fragments (0.5 μmol/L each) identified by SELEX-clos (Table 1) and SELEX-chip (Fig. 1 and Table 2) were subjected to gel-shift assay at increasing concentrations of RcdA protein (lanes 1–7: 0, 1.0, 2.5, 5.0, 10, 20, and 40 μmol/L). A total of seven representative targets, indicated on top of each gel, were examined. Even though all these test DNA probes exhibited RcdA dose-dependent formation of RcdA–DNA complexes, the number of complex bands differed because of the difference in the affinity to RcdA protein as estimated from the number of isolated clones by SELEX-clos (see Table 1) and the height of SELEX-chip peak (see Fig. 1 and Table 2). The ladders with decreased mobility include aggregates of RcdA–DNA complexes (see text for details).

The binding in vitro of purified RcdA to the sequences isolated by genomic SELEX screening was examined by PAGE analysis of RcdA–DNA complexes. Upon increase of RcdA addition, DNA fragments from all the major peaks of SELEX-chip formed multiple bands of RcdA–DNA complex (Fig. 3). The number of complex bands on PAGE, however, differed depending on the DNA probes, and correlated with the strength of RcdA binding as estimated from the number of isolated clones by SELEX-clos and the height of SELEX-chip peaks. DNA fragments that were not identified by genetic SELEX screening showed virtually no binding activity to RcdA (data not shown). The *ybjJ-rcdA* probe with the highest activity of RcdA binding generated more than 10 complex bands and upon further increase in RcdA concentration, the complexes migrated into a single big band. These observations altogether indicate that multiple molecules of RcdA bind to a single DNA probe and in addition, RcdA–DNA complexes assemble each other, leading to form aggregates of RcdA–DNA complexes. The strong cooperativity is similar to some nucleoid proteins such as H-NS (Shimada et al. 2009) and Dan (Teramoto et al. 2010), but is different from these nucleoid proteins with respect to the aggregate formation of DNA–protein complexes (see below for AFM images).

### AFM images of RcdA–DNA complexes

**Figure 4:**
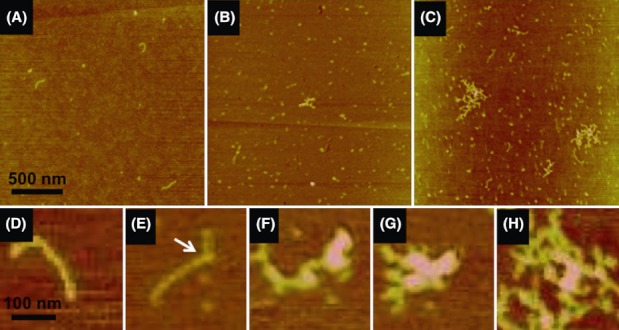
Atomic force microscopic (AFM) images of RcdA–DNA complexes. RcdA–DNA complexes formed using the spacer between *csgD*-*csgB* were observed with AFM. The DNA fragment was mixed with increasing concentrations of purified RcdA in the binding buffer (10 mmol/L Tris-HCl, pH 7.8, 3 mmol/L Mg acetate, 150 mmol/L NaCl, and 0.025 mg/mL BSA). After incubation for 30 min at 37°C, the RcdA–DNA mixture was subjected to AFM observation as described in Experimental Procedures. The RcdA/DNA molar ratio was: 0 (A), 1 (B), and 4 (C). Expanded images of some representative specimens with the increase in RcdA addition are shown in (D) to (H). An arrow in Figure 4E indicates RcdA protein associated at the initial binding site.

To confirm the predicted mode of RcdA–DNA interaction, we next tried to observe RcdA–*csgD* promoter DNA complexes with AFM. A 496-bp-long *ybjJ-rcdA* DNA fragment (see Fig. 1) was mixed with various concentrations of purified RcdA protein, and after 1-h incubation, the mixture was fixed by treatment with glutaraldehyde and subjected to AFM observation (Fig. 4). Under the conditions employed, the DNA probe attached with a single molecule of RcdA was rare even at low concentrations of RcdA (Fig. 4E), presumably because they are easily converted to DNA that are fully covered with RcdA (Fig. 4F–H). Upon increase of RcdA addition, the level of probe DNA fully covered with RcdA increased concomitant with the decrease in naked DNA (Fig. 4A–C). Both Dan and H-NS also cover entire DNA surface at high protein concentrations but in these cases, intermediate states of DNA–protein complexes could be observed (Teramoto et al. 2010; Walthers et al. 2011), implying that the cooperative interaction is much stronger for RcdA than Dan and H-NS. Upon further increase in RcdA concentrations, DNA–RcdA complexes were assembled to form irregular aggregates (Fig. 4G). At intermediate RcdA concentration, two types of RcdA–DNA complexes were observed, that is, single DNA probes with fully covered with RcdA (type-1) (Fig. 4F) and aggregates of type-1 RcdA–DNA complexes (type-2) (Fig. 4G). In the absence of glutaraldehyde, samples were easily dissociated from the mica film used in this study.

### Recognition sequence of RcdA

**Figure 5:**
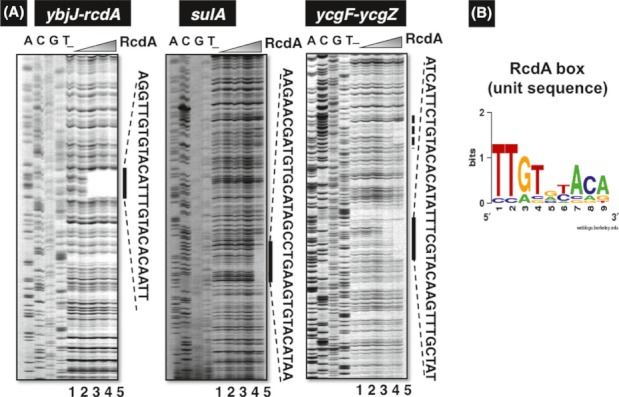
DNase-I footprinting analysis of RcdA-binding sequences. (A) DNase-I footprinting analysis of RcdA-binding sites was performed under the standard conditions (for details see Experimental Procedures) for the *ybjJ-rcdA* spacer (left panel), *sulA* ORF (center panel) and *ycgF-ycgZ* spacer (right panel) sequences. The RcdA concentrations used (left panel) were (from lane 1 to 5): 0 (lane 1), 0.1 (lane 2), 0.5 (lane 3), 1.0 (lane 4), and 5 pmol (lane 5). The sequences protected by RcdA from DNase-I digestion are shown on right side of each panel. The dotted line on left of *ycgF-ycgZ* fragment (right panel) includes 26-nucleotide-long junction sequence (5′GCTGTCGGAATGGACGA-TTGTATAGA3′) between the vector used for SELEX fragment cloning and the *ycgF-ycgZ* spacer, of which 5′-proximal 17-nucleotide sequence corresponds to the vector sequence whereas 3′-proximal 9-nucleotide sequence corresponds to *ycgF-ycgZ* sequence. Noteworthy is that the 3′-proximal sequence contains the RcdA box (TTGTnTACA)-like sequence, which might contribute the generation of footprinting. (B) In addition to these three DNase-I footprinting patterns, the protected sequences of *csgD-csgB*, *ydeI-ydeJ*, and *ynfM-asr* spacer regions by RcdA from DNase-I digestion were used for estimation of the consensus sequence of RcdA binding (data not shown). The logo pattern obtained using WebLogo (http://weblogo.berkeley.edu/logo.cgi) was defined as the RcdA-box unit sequence. The number and location of this unit sequence are different among the RcdA target genes (for details see text).

In order to identify the initial binding site of RcdA on the promoter regions of target genes, we performed DNase-I footprinting assay. At low RcdA concentrations, a single clear protection band was identified for the intergenic spacer region between ybjJ and rcdS (Fig. 5A, right panel). A clear protection bands was also identified within the intergenic region upstream of *sulA* (Fig. 5A, center panel). Within the intergenic spacer between *ycgF* and *ycgZ* (Fig. 5A, right panel), we identified two protection bands, the promoter-proximal 36-nucleotide-long band (indicated by a solid line) and the promoter-distal 26-nucleotide-long band (indicated by a dotted line) (Fig. 5A, right panel). The promoter-distal band includes the junction sequence between the 19-nucleotide-long cloning vector and the 9-nucleotide-long SELEX fragment. The RcdA box (TTGTnTACA)-like sequence was present in the latter fragment (see legend for Fig. 5). The RcdA-binding sequences were also identified in the *csgD-csgB*, *ydeI-ydeJ,* and *ynfM-asr* spacer regions (data not shown). Among all these RcdA-binding sequences, a consensus sequence of TTGTnnACA was identified, which we predicted to be the binding target sequence of RcdA, referred to RcdA-box (Fig. 5B). In the case of intergenic spacer between *ybjJ* and *rcdA*, the RcdA-box formed a palindromic sequence of TTGTGACAtttGTaCACAA (Fig. 5A, left panel). In contrast, this RcdA-box sequence is located in tandem on the *sulA* gene and within the *ycgF-ycgZ* spacer. The sequence length protected from DNase I digestion differed between the targets herein analyzed (Fig. 5A), reflecting the number and location of RcdA box.

### Regulatory role of RcdA in expression of the target genes

**Figure 6:**
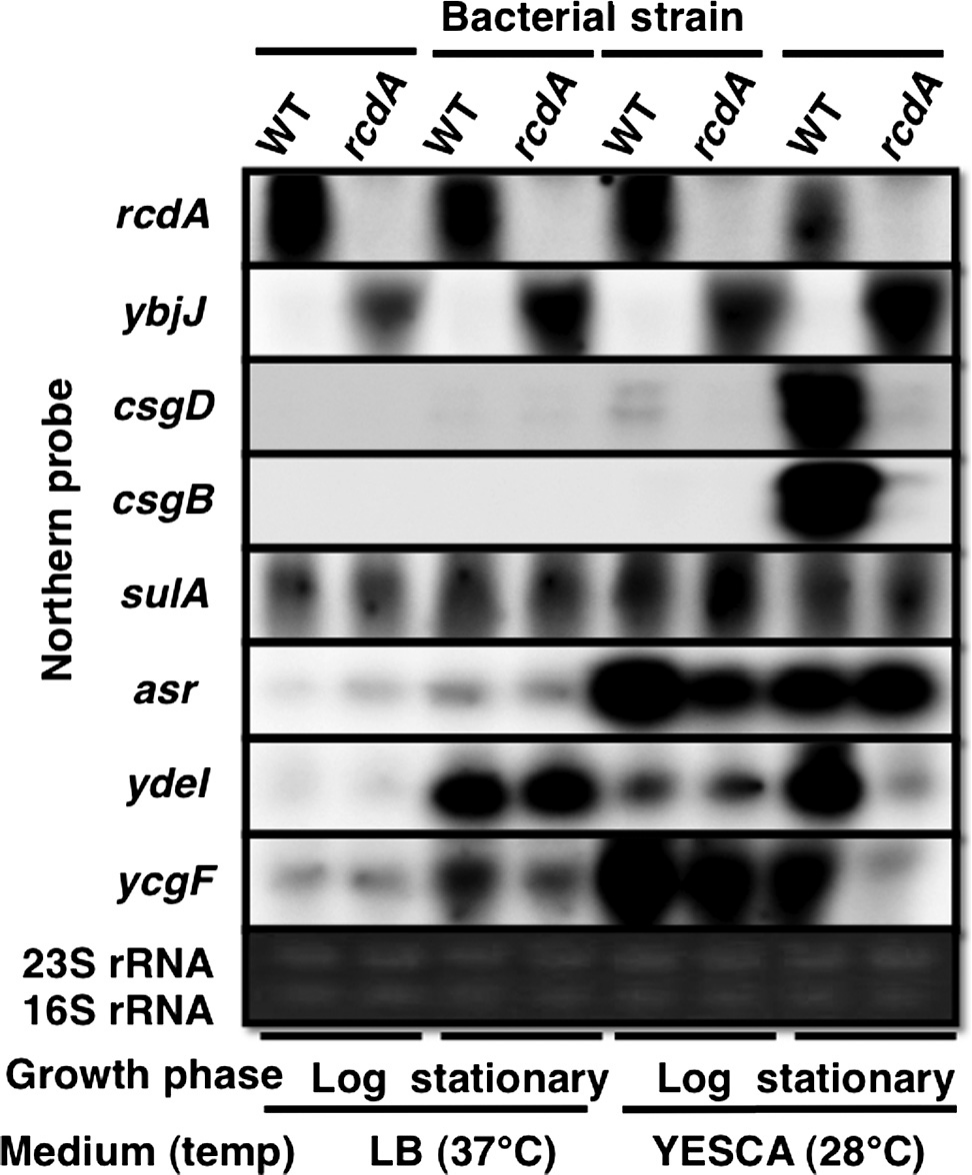
Northern blot analysis of RcdA target genes for wild-type and *rcdA* mutant. Wild-type *Escherichia coli* K-12 BW25113 and its *rcdA* mutant were grown in either LB at 37°C or YESCA at 28°C. Total RNA was prepared at both exponential phase and stationary phase, and immediately subjected to Northern blot analysis under the standard conditions (for details see Experimental Procedures). DIG-labeled hybridization probes are shown on left side of each panel. The amounts of total RNA analyzed were examined by measuring the intensity of ribosomal RNAs.

To examine possible influence of RcdA on the activity of promoters with RcdA-binding sites, we next performed Northern blot analysis for determination of mRNA levels for each of the predicted RcdA target genes in the presence and absence of RcdA (Fig. 6). In wild-type *E. coli*, *rcdA* mRNA was found to be expressed in both LB (37°C) and YESCA (28°C) and in both log and stationary phase (probe *rcdA* lane). Western blot analysis supported this conclusion and indicated detectable levels of RcdA protein (data not shown). On the other hand, the expression of the divergently transcribed *ybjJI* operon was expressed in the absence of RcdA (Fig. 6, *ybjJ* lane), indicating the involvement of RcdA as a repressor for the *ybjJI* operon.

The *csgD* gene for the master regulator of biofilm formation forms a single operon with *csgEFG* encoding the proteins for transport and assembly of curlin fimbriae. High-level expression of *csgD* was detected only in wild-type BW25113 grown at 28°C in YESCA medium, which is widely used for biofilm assay, but under the same culture condition, *csgD* mRNA was not detected for JW5114 lacking the *rcdA* gene (Fig. 6, *csgD* lane). Accordingly, expression of *csgB* that is under the positive control of CsgD was not detected (Fig. 6, *csgB* lane).

RcdA-binding site is located within *sulA* ORF, but its expression level was essentially the same between wild-type BW2113 and *rcdA* mutant JW5114. The *asr* gene encoding acid-shock protein was also expressed when cultivated at 28°C in YESCA medium, and its expression level significantly reduced in the *rcdA* mutant in particular at exponential growth phase (Fig. 6, *asr* lane). The stress-response protein YdeI was induced in stationary phase in both LB and YESCA media, but its expression was dependent on RcdA only cultivated in YESCA media (Fig. 6, *ydeI* lane). The *ycgF* gene encoding blue light-responsive regulator with EAL domain was also induced in stationary phase and its expression markedly decreased in the absence of RcdA (Fig. 6, *ycgF* lane). The Northern blot analysis clearly indicated the involvement of RcdA as a key and positive regulator of biofilm formation and response to various external stresses.

### Autogenous regulation of the *rcdA* gene

**Figure 7:**
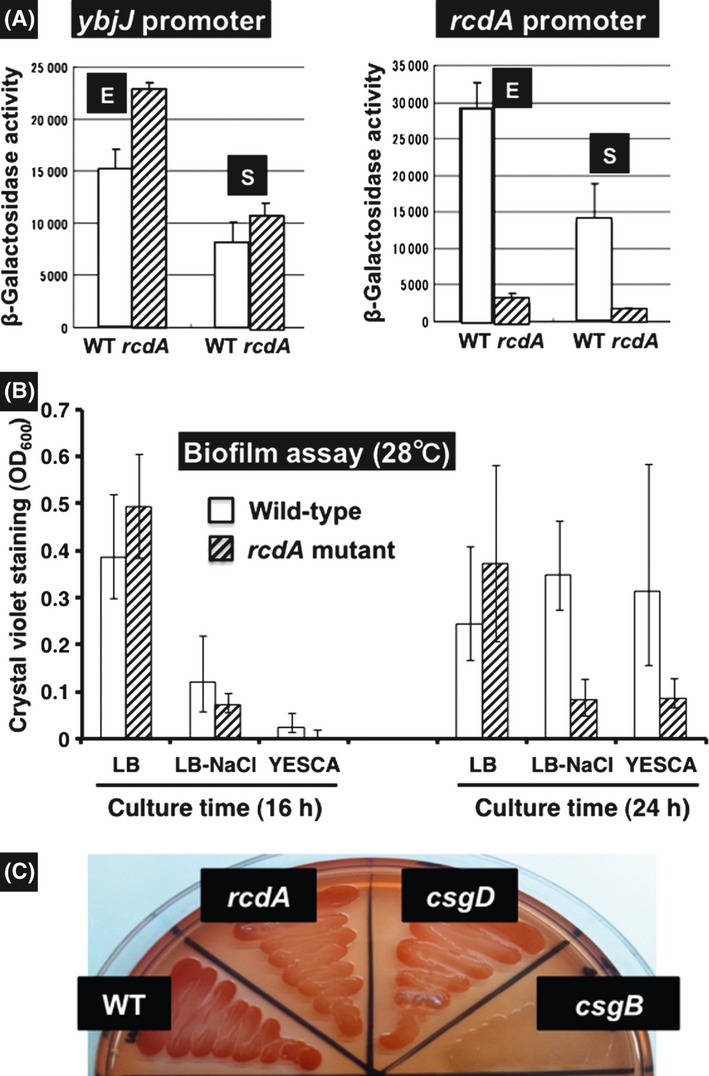
(A) Measurement of the activity of *ybjJ* and *rcdA* promoters. Promoters of the predicted regulation targets of RcdA were cloned into the promoter assay vector using LacZ reporter, and the resulting vectors were transformed into wild-type *Escherichia coli* BW25113 and otherwise isogenic *rcdA* mutant JW5114. The promoter activity was determined by measuring β-galactosidase activity. (B) The level of biofilm formation was measured by Crystal violet staining. *Escherichia coli* K-12 wild-type BW25113 and otherwise isogenic *rcdA* mutant JW5114 were grown in LB, LB plus NaCl, and YESCA media. The level of biofilm formation was measured at 16 (left panel) and 24 h (right panel) after inoculation. (C) The level of biofilm formation was measured with Congo red staining. *Escherichia coli* K-12 wild-type BMW25113 and the *rcdA* mutant JW5114 were grown on LB plate, and after 24 h, stained with Congo red.

As RcdA was suggested to play a key role in biofilm formation and stress responses, we next tested the regulatory mode of *rcdA* expression. Both Northern blot and Western blot analyses indicated a high-level expression of RcdA in wild-type *E. coli* strain throughout growth phases. To test the nature of *rcdA* promoter, we constructed an *E. coli* strain carrying the *rcdA*-*lacZ* transcriptional fusion on the genome, and performed the LacZ reporter assay in both wild-type and *rcdA* mutant strains. Marked decrease of LacZ level was observed for the *rcdA* mutant (Fig. 7A, *rcdA* promoter), indicating that transcription of *rcdA* is under the autogenous and positive control of RcdA. In contrast, the activity of *ybjJ* promoter for transcription toward opposite direction was rather increased in the *rcdA* mutant (Fig. 7A, *ybjJ* promoter). In addition to the *rcdA* promoter, significant decrease in the promoter activity was observed for the *appY* (regulator for anaerobiosis and stationary-phase adaptation)*, appC* (cytochrome *bd-*II terminal oxidase), and *cydA* (cytochrome *bd*-I terminal oxidase) promoters (data not shown), indicating that these promoters are also under positive control of RcdA.

### Regulatory role of RcdA in biofilm formation

CsgD is the master regulator of biofilm formation. After genomic SELEX screening, the *csgD* promoter was found to be a regulation target of RcdA. Involvement of RcdA as a key regulator of biofilm formation was then confirmed by using the Crystal violet staining assay of biofilm (Sule et al. 2009). For wild-type strain BW25113, biofilm is induced after prolonged incubation at low temperature and in YESCA media or in the presence of high concentrations of NaCl in LB medium (Fig. 7B, WT column). Under these conditions, the level of biofilm formation was markedly reduced for the mutant JW5114 lacking *rcdA* (Fig. 7B, *rcdA* column). Biofilm formation of wild-type, *rcdA, csgD,* and *csgB* mutants grown on YESCA plate at 28°C was also examined by staining with Congo red (Ogasawara et al. 2010a). The staining intensity by Congo red decreased to similar extent for both *csgD* and *rcdA* mutants (Fig. 7C). These findings support the conclusion that RcdA is involved in regulation of the *csgD* gene encoding the master regulator of biofilm formation.

**Figure 8:**
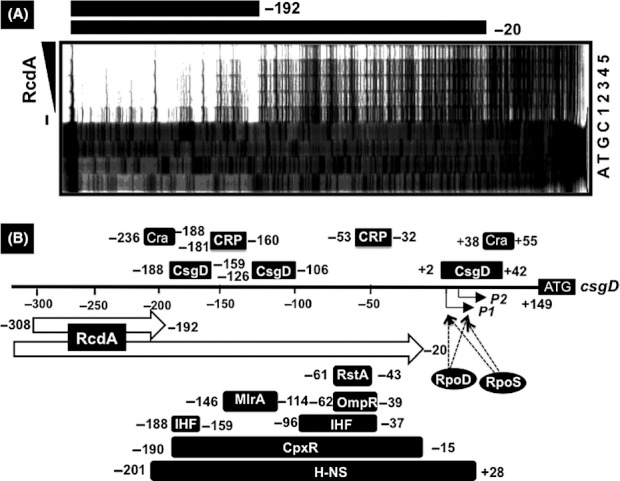
RcdA-binding site on the *csgD* promoter. (A) DNase-I footprinting assay of RcdA-binding site on the *csgD* promoter. DNase-I footprinting assay was performed for the spacer between *ybjJ* and *rcdA* under the standard reaction conditions as described in Figure 5. Upon increase of RcdA addition, the protected region from DNase-I digestion extended from −192 to −20. In this region, at least four RcdA-box-like sequences exist. (B) The location of transcription factor-binding sites on the *csgD* promoter. Previously, we determined the binding sites of Cra, CRP, CpxR, CsgD, H-NS, IHF, MlrA, OmpR, and RstA on the *csgD* promoter (Ogasawara et al. 2010a,b). Concomitant with the increase in RcdA concentration, its binding sequence expanded from the initial binding site so as to overlap the binding sequence by the universal silencer H-NS.

The *csgD* promoter is considered to be the most complex in *E. coli* as judged from the number of transcription factors participating in its regulation. More than 10 transcription factors, each monitoring a specific environmental stress condition, bind at less than 300-bp-long narrow regions of the *csgD* promoter for both positive and negative regulation of expression of the *csgD* operon (Fig. 8B) (Ishihama 2010; Ogasawara et al. 2010a,b). One of the unique features of this complex promoter is the collaboration between positive regulators and between negative regulators. We then determined the binding site of RcdA on the *csgD* promoter. By DNase-I footprinting assay, the initial binding site observed at low protein concentrations was found to be located within the sequence between −192 and −308, of the *csgD* promoter (Fig. 8A). As noted above, at least four RcdA-box-like sequences were detected in this region. Accordingly, the regions protected from DNase I digestion further expanded toward both upstream and downstream from this initial binding site. These observations altogether indicate that the RcdA protein is remarkably unique with respect to the cooperativity in protein–protein interaction when bound onto DNA.

## Discussion

The genomic SELEX screening system was developed for search of the recognition and binding sequences by DNA-binding transcription factors (Shimada et al. 2005) and successfully employed for identification of the whole sets of regulation targets by a number of transcription factors such as RstA (Ogasawara et al. 2007a), PdhR (Ogasawara et al. 2007b), LeuO (Shimada et al. 2009), CRP (Shimada et al. 2011b), Cra (Shimada et al. 2011c), and H-NS (Shimada et al. 2009). The genomic SELEX screening is in particular useful for search of regulation target genes under the direct control of uncharacterized putative transcription factors because of the lack of useful knowledge of phenotype screening. For instance, the regulatory roles of uncharacterized transcription factors YcdC, YdhM, and YgiP have been identified to be RutR (regulator of pyrimidine synthesis and degradation) (Shimada et al. 2007), NemR (*N*-ethylmaleimide reductase repressor) (Umezawa et al. 2008), and Dan (DNA-binding protein under anaerobic conditions) (Teramoto et al. 2010), respectively. In the course of our systematic search for the regulatory targets of about 100 species of the unknown transcription factors from *E. coli*, we found the involvement of YbjK as a regulator of *csgD* encoding the master regulator of biofilm formation, and renamed YbjK to RcdA (regulator of *csgD*). RcdA is one of the uncharacterized putative transcription factors belonging to the TetR family with a molecular size of 178 residues (Mr 20,307). In addition, a number of stress-response genes such as the genes for acid stress response, and response to blue light were found to be under the direct control of RcdA. We then realized that RcdA plays an important role in *E. coli* response to various stresses in nature, including biofilm formation.

CsgD (*C*urlin *s*ubunit *g*ene *D***)** is a FixJ/LuxR/UhpA-family transcription factor that regulates the *csgBAC* and *csgDEFG* operons for the synthesis, secretion, and assembly of curli components (Hammar et al. 1995; Loferer et al. 1997; Romling et al. 1998; Prigent-Combaret et al. 2001; Brombacher et al. 2003, 2006; Gualdi et al. 2008). The C-terminal domain of CsgD contains a potential helix-turn-helix DNA-binding motif whereas its N-terminal domain contains the receiver domain (Volz 1993). Besides the *csgBAC* and *csgDEFG* operons, CsgD is known to regulate *yaiC* (renamed to *adrA*) encoding the diguanylate cyclase for the synthesis of c-di-GMP (Chirwa and Herrington 2003; Pesavento et al. 2008), which plays dual roles, enhancement of biofilm formation and inhibition of cell motility through repression of flagella production and rotation (Wolfe and Visick 2008). Changes in various environmental conditions such as low osmolarity, low temperature, nutrient starvation, and high cell density influence, directly or indirectly, the expression of CsgD (Brombacher et al. 2003; Bougdour et al. 2004; Gualdi et al. 2008). Reflecting the responsibility of CsgD expression to various external stresses, more than 10 different but known transcription factors, each monitoring a different environmental factor or condition, are involved in the regulation of *csgD* promoter (Ishihama 2010; Ogasawara et al. 2010a,b). By using the newly developed PS-TF (promoter-specific transcription factor) screening system, several other members of both known and unknown transcription factors have been suggested to be involved in direct control of the *csgD* promoter (A. Ishihama et al., unpubl. data).

One of the unique features of RcdA is its mode of DNA binding. By gel-shift assay, it formed multiple bands of DNA complexes as in the case of Dan (Teramoto et al. 2010). Dan binds to DNA in a polymerization or stiffening mode, leading to form Dan–DNA rods at saturation. The initial binding site of RcdA on the *csgD* promoter is located within the approximately 110-bp-long sequence between −308 and −192 upstream of two hot spots of the contact sites of more than 10 transcription factors (see Fig. 8B). Upon increase of RcdA concentration, its binding expands from the initial binding site (between −308 and −192) down to −20 within the promoter sequence. The entire RcdA-binding region largely overlaps with the H-NS-binding region (−201 and +28). AFM analysis of RcdA–DNA complexes indicated that in addition to polymerization along a single DNA probe, RcdA induces the association of RcdA–DNA complexes through bridging (polymerization mode), forming aggregates of RcdA–DNA complexes (aggregation mode) (see Fig. 4). Two modes of DNA binding are similar to H-NS (Amit et al. 2003), which plays a key role in xenogenetic silencing of horizontally acquired genes. In the case of H-NS, antisilencers remove H-NS only when bound in polymerization mode (Fang and Rimsky 2008), indicating that two modes of DNA binding represent different role of H-NS. Taken together, we suggest one possible mechanism of *csgD* promoter activation by RcdA is its antisilencing role for displacement of the general silencer H-NS that covers the entire *csgD* promoter region including both P1 and P2 promoters for recognition by RpoD and RpoS RNA polymerases (Ogasawara et al. 2010a,b) (see Fig. 8B).
